# Correction: Transcriptomic analysis of the developing and adult mouse cochlear sensory epithelia

**DOI:** 10.1371/journal.pone.0240731

**Published:** 2020-10-13

**Authors:** Ibtihel Smeti, Said Assou, Etienne Savary, Saber Masmoudi, Azel Zine

## Notice of republication

This article [1] was republished on September 30, 2020, to remove an image which was not offered under a CC-BY license.

Similarities were noted between images presented in this article [1] and two additional articles published by the same research group [2, 3]:

- The schematics of postanal day-3 and adult cochlear sensory epithelia presented in Fig 1 [1] are identical to the schematics of postanal day-3 and adult cochlear sensory epithelia presented in Fig 1A [2] and the schematics presented in *Gene Expression Patterns* [3].
- The immunohistochemistry images of undifferentiated CGR8 mouse embryonic stem cells in Fig 4D–4F [1] are identical to the immunohistochemistry images of undifferentiated CGR8 mouse embryonic stem cells in Fig 1B [2].

The authors indicated that the schematics presented in the *PLOS ONE* articles [1, 2] are adapted from the schematic published in *Gene Expression Patterns* [3], which is not offered under a CC-BY license. As the original Fig 1A is not licensed for reproduction and distribution under the terms of the Creative Commons Attribution License (or Public Domain License for US gov), this content has been removed from the republished article and replaced with an alternative relevant schematic image. Please download this article again to view the correct version.

In addition to the updated figure, additional items were clarified in the republished article.

Specifically,

- The immunohistochemistry images presented in Fig 4D–4F [1] and Fig 1B [2] represent the same positive control for HMAG2 immunostaining. The authors indicated that the positive controls presented in these figures were not conducted simultaneously with the immunohistochemistry experiments reported in the articles but have been included for reference only, which has been clarified in the updated figure legend.
- The authors have provided further details regarding the methodology of their cell culturing, tissue preparation, and immunohistochemistry experiments. The following information has been added to the Materials and Methods section:

**“Culture of mouse embryonic stem cells (mESCs)**
  The undifferentiated mESCs (CGR8 line kindly provided by Bernard Binetruy, Aix-Marseille University, France) were expanded in the absence of feeder cells in DMEM culture medium (Gibco by Life Technologies) supplemented with LIF (leukemia inhibitory factor) on gelatin-coated plates. When the propagated cells were confluent at 80–90% (about 5–7 days), they were passaged using 0.25% trypsin-EDTA (Gibco by Life Technologies). The undifferentiated and untreated cells used for immunohistochemistry were harvested from passage 2 cell cultures and fixed in paraformaldehyde 4% in Phosphate Buffer Solution (PBS) for 20 min at room temperature. After several rinses they were processed for immunohistochemistry with HMGA2 antiserum following the same immunostaining protocol used for cochlear tissue sections.
**Cochlear tissue preparation and Immunohistochemistry**
  For postnatal day-3 (P3) cochlear tissue, the animals were decapitated and their cochleas were removed and immersed in 4% paraformaldehyde in phosphate-buffered saline (PBS) at 4°C overnight. Next, the tissue was washed in PBS, and incubated on ice in 10%, sucrose solution for 0.5 h, and then incubated in 20% sucrose at 4°C overnight. The tissue was then incubated in 1:1 Cryo-OCT Compound (VWR, France) and 20% sucrose at 4°C overnight. Then, the tissue was washed in 100% OCT for 10 min and then frozen, cryosectioned in 10 um sections, and subsequently stored at -20°C.
  For labeling experiments, the tissue cryosections were incubated in PBS supplemented with 4% normal BSA plus 0.3% Triton X-100 (Sigma, France) for 2 h. Next the cryosections were incubated in a solution containing 1:200 dilution of rabbit antiserum anti-Hmga2 (Kind gift of Dr. Narita, Cambridge Research Institute, UK). Then, the sections were washed, and then incubated in anti-rabbit AlexaFluor-568 (Molecular Probes) diluted 1:500 in PBS for 2 h at RT and the nuclei were counterstained with Dapi. The sections were washed, then mounted using fluorescence mounting medium (Dako) and images were analyzed using DMRB fluorescence microscope (Leica).”

- The acknowledgments section has been updated to correct a spelling error in the name of the individual who donated the CGR8 cells. The correct sentence now reads:
  “We thank Drs T. Van de Water (University of Miami) for his critical reading, B. Binetruy (University Aix Marseille) for the ES CGR8 cells, M. Narita (Cambridge Institute) for the HMGA2 antibody and A. Dos Santos (UMR 7260, University Aix Marseille) for cell culture.”
- The authors have indicated that in addition to data within the paper and its Supporting Information files additional data are accessible at the gene expression Omnibus (GEO) repository https://www.ncbi.nlm.nih.gov/geo through the provisional accession series number GSE32963. The authors have stated that all the data underlying this study are still available upon request.

## Supporting information

S1 File Originally published, uncorrected article with copyrighted image removed. (PDF)
