# Supplementary figures and images for: Transcriptomic Analysis of the Developing and Adult Mouse Cochlear Sensory Epithelia
Source: PLoS One. 2012 Aug 10;7(8):e42987. doi: 10.1371/journal.pone.0042987 (PMC3416779; doi:10.1371/journal.pone.0042987)

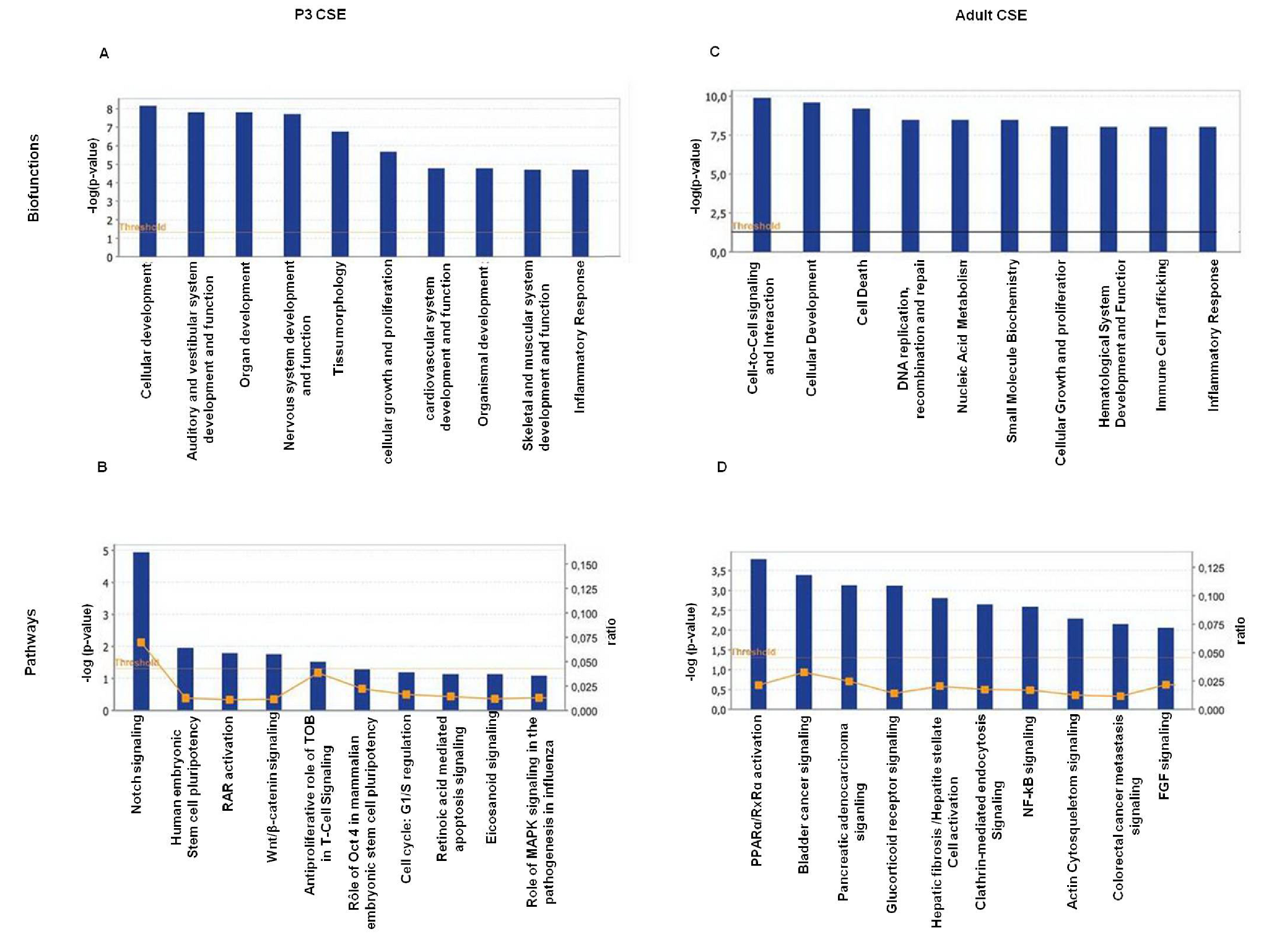

Supplement: Figure S1 — Biological functions and pathways associated with the selected differentially expressed genes. (A) IPA function analysis on the up-regulated P3 genes. (B) IPA pathway analysis on the up-regulated P3 genes. (C) IPA function analysis on the up-regulated adult genes. (D) IPA Pathways analysis on the up-regulated adult genes. The Threshold represents the p-value = 0.05. The ratio (shown as squares on B and D) represents the number of analyzed genes in a given pathway divided by total number of genes that form this pathway. (TIF) [file pone.0042987.s001.tif]

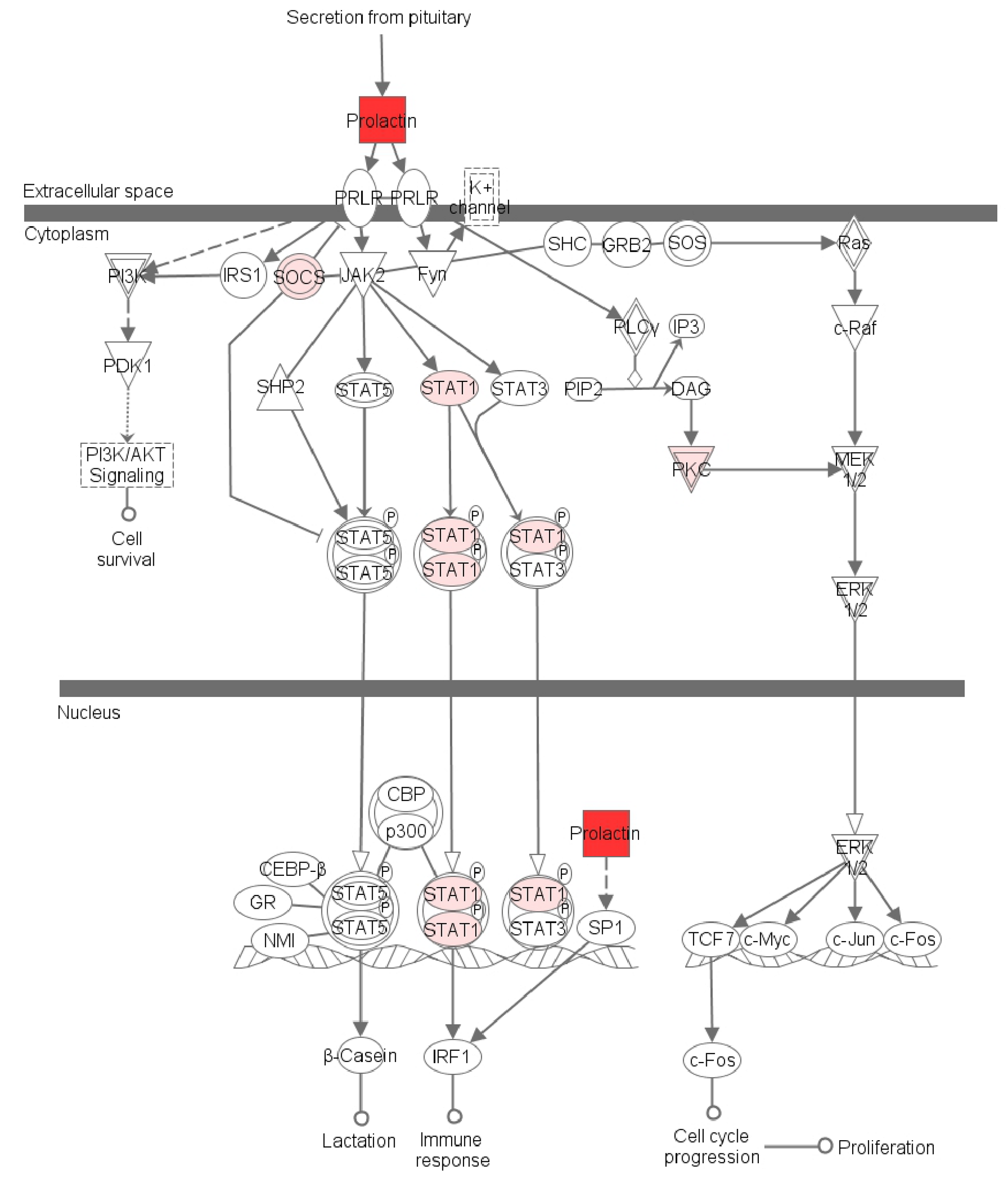

Supplement: Figure S2 — Prolactin signaling pathway identified by IPA software. The Prl transduction signal via PRLR shows that Prl may act via Jak2-Stat and/or Ras pathways depending on the function. Jak2-Stat pathway involves Stat1, Stat3 and Stat 5 transcription factors. Ras pathway is activated in cell cycle, proliferation and cell death functions. It involves Ap1 complex in the nucleus formed by c-Jun and c-Fos immediate genes. The pathway figure explains the interactions between Prl, Stat and AP1 shown in the IPA up adult network (Fig. 5). (TIF) [file pone.0042987.s002.tif]

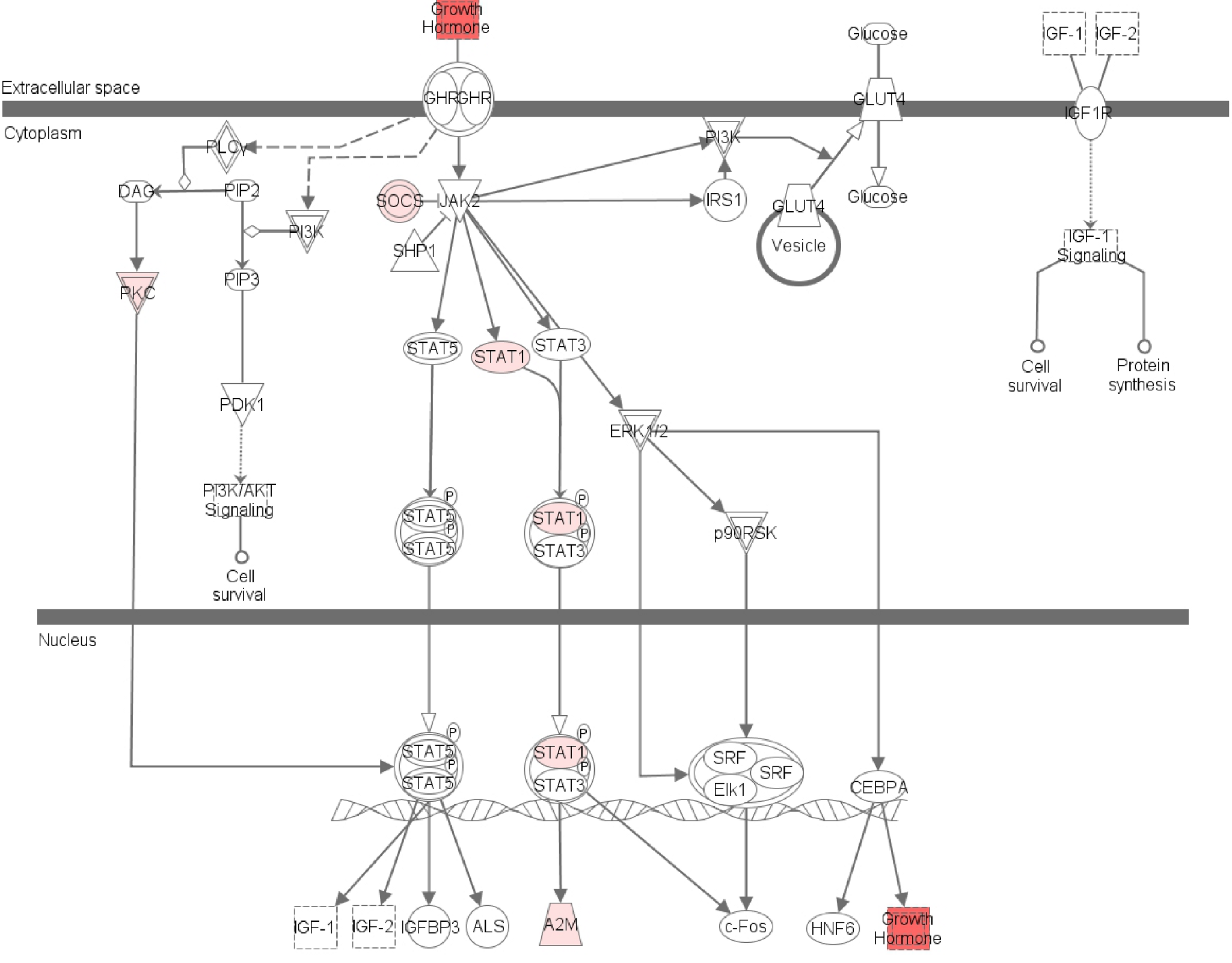

Supplement: Figure S3 — Growth hormone signaling pathway identified by IPA software. Canonical pathway reveals growth hormone-related gene signaling. Canonical pathway analysis with IPA software showing known growth-hormone related pathways including SOCS, STAT1 transcription factor, and PKC genes. The canonical pathway is established by the IPA software from its previous database of publications and may include computationally generated networks from many tissues at any age. Pink-colored genes are up-regulated genes in the adult list. (TIF) [file pone.0042987.s003.tif]

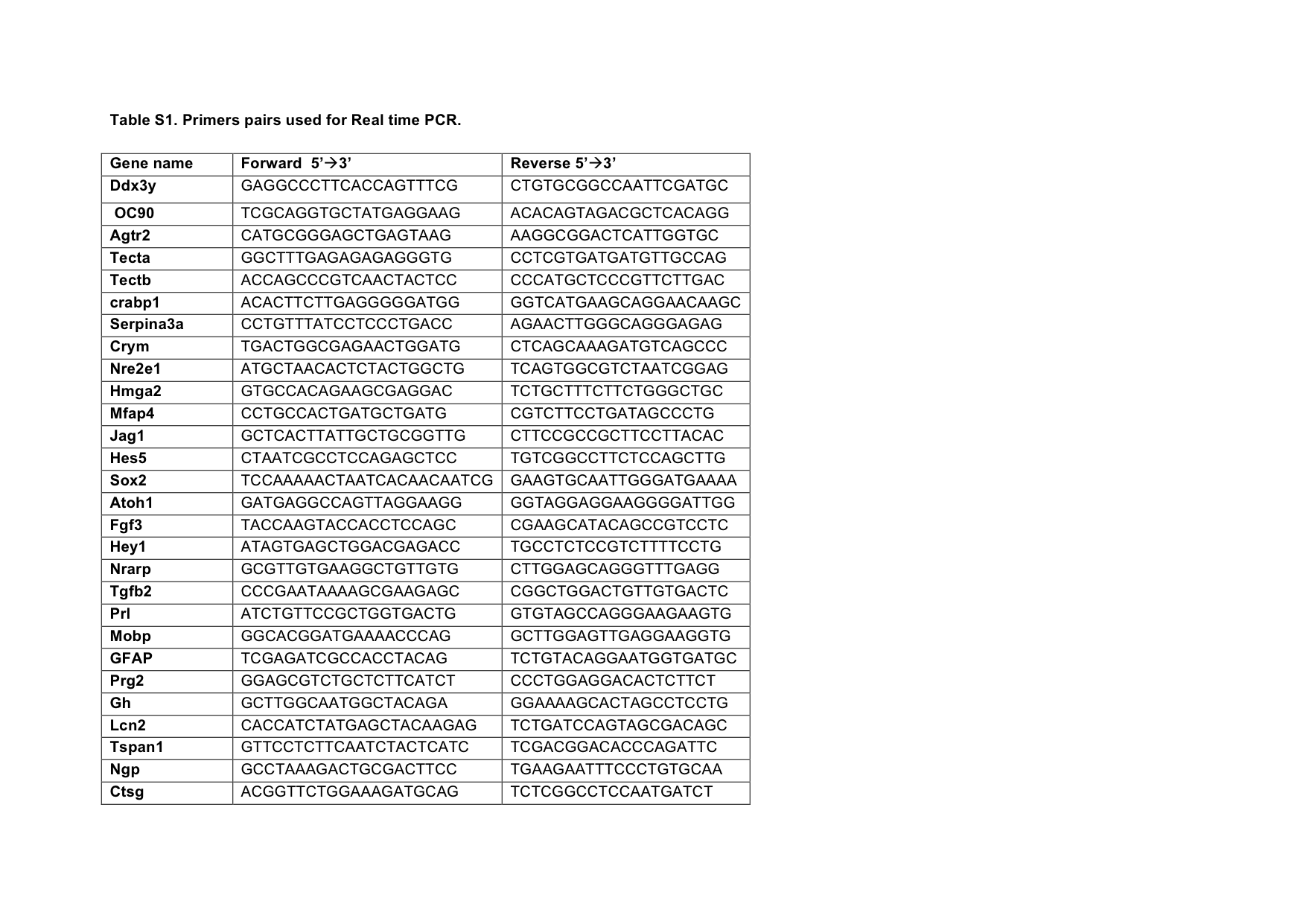

Supplement: Table S1 — Primers pairs used for Real time PCR. (TIFF) [file pone.0042987.s004.tiff]
