# Supplementary material for: Correction: Transcriptomic analysis of the developing and adult mouse cochlear sensory epithelia
Source: PLoS One. 2020 Oct 13;15(10):e0240731. doi: 10.1371/journal.pone.0240731 (PMC7553330; doi:10.1371/journal.pone.0240731)
Supplement: S1 File — (PDF) [file pone.0240731.s001.pdf]

Transcriptomic Analysis of the Developing and Adult Mouse Cochlear Sensory Epithelia

Ibtihel Smeti[1,2,4], Said Assou[3], Etienne Savary[1], Saber Masmoudi[4], Azel Zine[1,2]*

1 Integrative and Adaptative Neurosciences, UMR 7260 Aix-Marseille University/CNRS, France, **2** Sensory Biophysics, Faculty of Pharmacy, Montpellier I University, Montpellier, France, **3** CHU Montpellier, Institute for Research in Biotherapy, Inserm U 1040, Montpellier I University, Montpellier, France, **4** Laboratoire de Microorganismes et de Biomolécules, Centre de Biotechnologies, Sfax, Tunisie

Abstract

The adult mammalian cochlea lacks regenerative ability and the irreversible degeneration of cochlear sensory hair cells leads to permanent hearing loss. Previous data show that early postnatal cochlea harbors stem/progenitor-like cells and shows a limited regenerative/repair capacity. These properties are progressively lost later during the postnatal development. Little is known about the genes and pathways that are potentially involved in this difference of the regenerative/repair potentialities between early postnatal and adult mammalian cochlear sensory epithelia (CSE). The goal of our study is to investigate the transcriptomic profiles of these two stages. We used Mouse Genome 430 2.0 microarray to perform an extensive analysis of the genes expressed in mouse postnatal day-3 (P3) and adult CSE. Statistical analysis of microarray data was performed using SAM (Significance Analysis of Microarrays) software. We identified 5644 statistically significant differentially expressed transcripts with a fold change (FC) >2 and a False Discovery Rate (FDR) ≤ 0.05. The P3 CSE signature included 3,102 transcripts, among which were known genes in the cochlea, but also new transcripts such as, Hmga2 (high mobility group AT-hook 2) and Nrarp (Notch-regulated ankyrin repeat protein). The adult CSE overexpressed 2,542 transcripts including new transcripts, such as Prl (Prolactin) and Ar (Androgen receptor), that previously were not known to be expressed in the adult cochlea. Our comparative study revealed important genes and pathways differentially expressed between the developing and adult CSE. The identification of new candidate genes would be useful as potential markers of the maintenance or the loss of stem cells and regenerative/repair ability during mammalian cochlear development.

Citation: Smeti I, Assou S, Savary E, Masmoudi S, Zine A (2012) Transcriptomic Analysis of the Developing and Adult Mouse Cochlear Sensory Epithelia. PLoS ONE 7(8): e42987. doi:10.1371/journal.pone.0042987

Editor: Richard Libby, University of Rochester, United States of America

Received March 14, 2012; **Accepted** July 16, 2012; **Published** August 10, 2012

Copyright: © 2012 Smeti et al. This is an open-access article distributed under the terms of the Creative Commons Attribution License, which permits unrestricted use, distribution, and reproduction in any medium, provided the original author and source are credited.

Funding: Funding was provided by the ANR (Agence Nationale de Recherche; ANR-2010-BLAN-1107-01-b to AZ), The Fondation de France Fonds, and the Averroes program/University Montpellier I (doctoral fellowship to IS). The funders had no role in study design, data collection and analysis, decision to publish, or preparation of the manuscript.

Competing Interests: The authors have declared that no competing interests exist.

* E-mail: azel.zine@univ-montp1.fr

Introduction

Cochlear sensory epithelium (CSE) contains the auditory receptors refered to as hair cells (HCs) that are essential for hearing [1]. These sensory cells can be damaged as a consequence of acoustic trauma, ototoxic drugs, or simply with aging. Although, HCs in mammals are produced only during embryonic development and not able to regenerate when lost during the postnatal period of maturation [2], some studies using *in vitro* assay suggested a limited non-proliferative regeneration/repair capacity within the ototoxic-damaged explants derived from the early postnatal CSE [3–5]. It has been also suggested from studies using transgenic and knock-out mice [6–10] that some proliferative potential, although restricted under normal *in vivo* conditions, is retained in the early postnatal CSE. In addition, recent studies demonstrated the presence of stem/progenitor cells within the postnatal-P3 mouse CSE and their mitotic capacity to form clonal spheres when maintained under appropriate *in vitro* conditions [11–14]. Nevertheless, this stem cell population is progressively exhausted during later postnatal development [12]. Recently, we also showed that the supporting cells in the mouse postnatal CSE express many stem/progenitor markers which were down regulated in the adult, that could be correlated to the loss of stem/progenitor cells within the adult mammalian cochlea [14]. Thus, comparison of expression profiles between P3 and adult mouse CSE is hypothesized to identify differentially regulated genes involved in stem/progenitor cell maintenance and the capacity of this sensory epithelium for regeneration/repair.

DNA microarray is a powerful technology that currently permits comparison of gene expression at the whole-genome scale [15]. Gene expression profiling using microarrays has been applied in the inner ear during the past decade, especially in bird, fish and rodent species. Gene expression analysis within the bird inner ear was especially investigated in order to understand the molecular mechanisms that control the regeneration capacity [16,17], and also to gain insights on the genetic programs that control inner ear development [18]. In zebrafish, microarrays were applied to investigate the specific transcriptome of HCs [19]. Recently, one transcriptional analysis on zebrafish inner ear after acoustic trauma revealed growth hormone, as critically involved in the post-trauma regeneration process [20]. In mammals, gene expression profiling was investigated in order to identify tissue specific genes and/or to examine changes in gene expression under several conditions [21–25]. Regarding the gene expression changes in the mammalian cochlea during maturation, only a limited number of studies have been performed. Chen and

Corey [26], used GeneChip arrays (i.e., oligonucleotide array set covering 13,000 known genes and 21,000 EST clusters) to explore the gene expression patterns in whole cochlea between two developmental stages (i.e., P2 and P32) reporting a differential gene expression that correlates with the onset of cochlear function. In our study, we compared, for the first time, the whole genome expression profiles between the postnatal P3 and adult cochlea stages, using mouse chip Affymetrix 430.02.

The aim of this study is to explore changes in genes and pathways underlying the known difference in the stem/progenitor cells maintenance and in the capacity of regeneration/repair between P3 and adult CSE.

Materials and Methods

Ethics Statement

All animal work was conducted according to the Guide to the Care and Use of Laboratory Animals [27] and all procedures were approved by ethics Committees of the INSERM (Institut National de la Santé et de la Recherche Medicale) and CNRS (Centre National de la Recherche Scientifique).

Sample Collection and RNA Extraction

RNA samples used in this study were extracted from CSE dissected from postnatal day three (P3) and eight-week-old adult Swiss Webster mice. We used this mouse strain because it keeps normal hearing beyond eight weeks [28]. P3 mice were sacrificed by decapitation. For adult stage, cervical dislocation was performed before decapitation. The heads were hemisected along the sagital midline, and the brains removed. Cochleae were dissected from the temporal bones. After removing the otic capsule, the modiolus, the spiral ligament and the stria vascularis from the cochlea, the CSE were collected and immediately placed in RNA later solution (Ambion). For each stage (P3/adult), three independent dissection experiments were carried out separately in order to obtain three biological replicates. In each experiment, 40 CSE were extracted from 20 mice and pooled for RNA extraction. Thus, three independent RNA samples were obtained for each stage. The total RNA from each biological sample was extracted using RNAeasy Mini Kit (Qiagen, Valencia, CA) according to the manufacture's protocol. After several washings and DNase treatment, elution was carried out in 20 µL of DNase-free water. RNA was quantified using a Nanodrop ND-1000 spectrophotometer (Nanodrop Technologies, Wilmington, DE). RNA integrity and quality were evaluated with an Agilent 2100 Bioanalyzer (Agilent, Palo Alto, CA). RNA samples were stored at −80°C until microarray analysis.

Microarray Hybridization

The Affymetrix 3′ IVT express protocol was used to prepare cRNA (one-cycle amplification) with a starting concentration of 200 ng of total RNA. First-strand DNA was synthesized using an oligo-dT primer that incorporates a T7 promoter sequence. cDNA was then amplified by *in vitro* transcription (IVT) with T7 RNA polymerase. 12 µg of Biotinylated and fragmented cRNA were used for hybridization with Mouse Genome 430 2.0 gene chip (Affymetrix) that contains more than 45.000 transcripts covering the whole mouse genome. Subsequent washing and staining of the arrays were performed using the GeneChip Affymetrix station protocol. A total of six chips (three chips for P3 samples and three chips for adult samples) were used for microarray hybridization experiments. The microarray data were obtained in agreement with the minimal informations about microarray experiment (MIAME) recommendations (PMID: 11726920). All data are accessible at the gene expression Omnibus (GEO) repository https://www.ncbi.nlm.nih.gov/geo through the provisional accession series number GSE32963.

Data Analysis and Visualization

Scanned GeneChip images were processed using the Affymetrix GCOS 1.4 software. Microarray data were analyzed using the Affymetrix Expression Console^{TM} software and normalization was performed with the MAS5.0 algorithm to obtain the signal intensity and the detection call (present, marginal, or absent) for each probe set. To compare the gene expression profiles of the six CSE samples according to the P3 and adult stages, we first filtered the samples based on the "detection call". This "call" can either be "present" (when the perfect match probes are significantly more hydridized that the mismatch probe, $p<0.4$) "marginal" (for $p\geq0.04$ and ≤0.06) or absent ($p>0.06$). Probe sets were used when they were present in at least 4 samples out of 6. A statistical technique called Significance Analysis Microarrays (SAM) software [29] was used with a fold change of 2 and a false discovery rated (FDR) of <5%. SAM allowed the identification of genes whose expression varied significantly among the P3 and adult groups. Hierarchical clustering was carried out with CLUSTER and TREEVIEW software [30]. This algorithm is based closely on the average-linkage method of Sokal and Michener [31] based on correlation coefficient.

Quantitative Real Time PCR

We performed qRT-PCR to validate the expression of a set of selected genes. Primers used were designed with Primer Explorer 2.0 software or the online Roche software (www.universalprobelibrary.com, Roche). The GAPDH gene was used as endogenous control in order to normalize data. Each RNA sample was converted to cDNA using Random hexanucleotide primers and M-MLV reverse transcriptase (Invitrogen). The PCR reaction mix was prepared by adding QuantiTect SYBR Green PCR Master Mix at 1x, adequate primer at 0.5 µM and cDNA diluted at 1/25 to RNA free water. Amplification was performed using the Light Cycler 480 system (Roche) under the following conditions: an initial denaturation step at 95°C for 15 min followed by 45 three segment cycles of amplification (95°C/15 sec, 55°C/20 sec and 72°C/25 sec). For each target gene, three reactions were done against the endogenous control on the same run. The results were read using the Light Cycler 480 Software (Roche) and then analyzed using the delta Ct ΔCt method [32]. The list of primer sequences is shown in Table S1.

Statistical Analysis

We used Mann Whitney test from GraphPad Software to analyze the RT-per data. The differences among cochlear samples were considered significant when the p-value was ≤0.05.

Gene Ontology Enrichment Analysis

The gene ontology (GO) enrichment analysis, the biological processes and networks of the differentially expressed genes were generated by Ingenuity Pathway Analysis (IPA) tools (www.ingenuity.com). The 46 genes (Tables 1 and 2) were imported into IPA and each gene identifier was overlaid onto a global molecular network developed from information contained in the Ingenuity Pathways Knowledge Base. Networks of these genes were then generated based on their connectivity. A network score was calculated based on the hyper-geometric distribution and calculated with the right-tailed Fisher's exact test (P value <0.05 was considered significant). The over-representation of functional

Table 1. The 20 selected P3 up-regulated genes.

Probeset	UniGene ID	Gene Title	Gene Symbol	Fold change P3/ Adult
1452403_a_at	Mm.41776	otoconin 90	Oc90	131.29
1415832_at	Mm.2679	angiotensin II receptor, type 2	Agtr2	107.91
1419632_at	Mm.42209	tectorin alpha	Tecta	73.56
1430912_a_at	Mm.42139	tectorin beta	Tectb	59.68
1448326_a_at	Mm.34797	cellular retinoic acid binding protein I	Crabp1	57.98
1421556_at	Mm.159128	serine (or cysteine) peptidase inhibitor, clade A, member 3A	Serpina3a	48.49
1416776_at	Mm.9114	crystallin, mu	Crym	45.35
1434921_at	Mm.287100	nuclear receptor subfamily 2, group E, member 1	Nr2e1	39.33
1422851_at	Mm.157190	high mobility group AT-hook 2	Hmga2	26.75
1456883_at	Mm.39821	storkhead box 1	Stox1	26.46
1417359_at	Mm.7386	microfibrillar-associated protein 2	Mfap2	20.79
1424010_at	Mm.272278	microfibrillar-associated protein 4	Mfap4	16.85
1421106_at	Mm.22398	jagged 1	Jag1	7.02
1423146_at	Mm.137268	hairy and enhancer of split 5 (Drosophila)	Hes5	5.76
1416967_at	Mm.65396	SRY-box containing gene 2	Sox2	5.42
1449822_at	Mm.57229	atonal homolog 1 (Drosophila)	Atoh1	5.07
1441350_at	Mm.4947	fibroblast growth factor 3	Fgf3	4.29
1415999_at	Mm.29581	hairy/enhancer-of-split related with YRPW motif 1	Hey1	3.87
1417985_at	Mm.46539	Notch-regulated ankyrin repeat protein	Nrarp	3.67
1450922_a_at	Mm.18213	transforming growth factor, beta 2	Tgfb2	3.16

doi:10.1371/journal.pone.0042987.t001

categories, biological processes and canonical pathways of the Q-PCR validated genes is confirmed by the gene annotation web tool "DAVID", Database for Annotation, Visualization and Integrated Discovery (http://david.abcc.ncifcrf.gov/).

Results

Cochleae were removed from P3 and adult mice. The stria vascularis, Reissner's membrane, spiral ligament and a major part of the spiral ganglion were removed. The remaining cochlear tissue is designated in this study as micro-dissected cochlear sensory epithelia (CSE). It consists of two types of sensory hair cells (inner and outer) and four types of supporting cells (Dieter's, Hensen's, Claudius', inner and outer pillar cells), in addition to supporting cell subtypes (border and inner phalengeal cells) within the inner hair cell area. In the P3 cochlea, micro-dissected CSE samples included also thickened areas formed by tall columnar cell mounds, i.e. the greater epithelial ridge (GER) and the lesser epithelial ridge (LER). During development the GER cells progressively recede to form the inner sulcus (IS). In the adult cochea, the micro-dissected CSE samples consists of two types of hair cells (inner and outer) and four types of supporting cells (Deiters's, Hensen's, Claudius', inner and outer pillar cells), in addition to the IS and interdental cells within the limbus zone (Fig. 1). In all cases, the tissue micro-dissections were performed by the same approach and by the same investigator and special care was taken to keep, as much as possible, all parameters constant.

Identification of Differentially Expressed Genes between P3 and Adult CSE

We were interested in the changes in gene regulation within the CSE that occurred between P3 and adult stages. Using the SAM analysis, we identified a total of 5644 transcripts with a False Discovery Rate (FDR) $\leq$0.05 that significantly distinguished the P3 and adult CSE sample groups. Among those transcripts, 3102 are up-regulated in P3 (i.e., P3 molecular signature), in contrast the remaining 2542 are preferentially regulated in the adult (i.e., adult molecular signature). The number of transcripts that are specific for a given category of a CSE sample indicates that there is a significant variation across the two categories of CSE as demonstrated also by the hierarchical clustering which shows a clear segregation of the CSE samples based on this list of 5644 transcripts (Fig. 2). The six samples that we analyzed are arranged into two major clusters as shown on the top of the dendrogram (Fig. 2). Every three CSE samples corresponding to the same stage form a main cluster together. This result indicates that the triplicate samples for the same condition are concordant, supporting the reliability of our microarray assay. However, within the P3 samples cluster, the samples 2 and 3 are more related to each other than sample 1. In addition, the samples 1 and 3 in adult samples cluster are more linked than sample 2. These differences could be related to variations of the sample collection during the dissection procedure.

Validation of the Microarray Results

Among SAM transcripts, we selected 38 genes to validate by Q-PCR. These genes were chosen based either on their high fold change and/or their potential functions. These genes are named "Top 20 P3 up-regulated genes (Top up P3)" (FC ranged from 3.16 to 131.29) and "Top 26 up-regulated adult genes (Top up adult)" (FC ranged from 2.36 to 460.06), and listed in Tables 1 and 2 respectively. Both top up P3 and top up adult lists contain known and previously characterized genes

Table 2. The 26 selected adult up-regulated genes.

Probeset	UniGene ID	Gene Title	Gene Symbol	1/fold change P3/ Adult
1429287_a_at	Mm.1270	prolactin	Prl	460.06
1433785_at	Mm.40461	myelin-associated oligodendrocytic basic protein	Mobp	334.96
1426509_s_at	Mm.1239	glial fibrillary acidic protein	Gfap	119.84
1422873_at	Mm.142727	proteoglycan 2, bone marrow	Prg2	104.55
1460613_x_at	Mm.343934	growth hormone	Gh	89.18
1427747_a_at	Mm.9537	lipocalin 2	Lcn2	63.43
1417957_a_at	Mm.45994	tetraspanin 1	Tspan1	58.1
1418722_at	Mm.236225	neutrophilic granule protein	Ngp	45.65
1419594_at	Mm.4858	cathepsin G	Ctsg	38.3
1417933_at	Mm.358609	insulin-like growth factor binding protein 6	Igfbp6	32.85
1456944_at	Mm.249386	potassium voltage gated channel, Shaw-related subfamily, member 1	Kcnc1	28.15
1437672_at	Mm.24486	insulin receptor substrate 3	Irs3	27.18
1435094_at	Mm.30176	potassium inwardly-rectifying channel, subfamily J, member 16	Kcnj16	20.85
1417262_at	Mm.292547	prostaglandin-endoperoxide synthase 2	Ptgs2	12.26
1416957_at	Mm.897	POU domain, class 2, associating factor 1	Pou2af1	7.56
1423136_at	Mm.241282	fibroblast growth factor 1	Fgf1	6.89
1440270_at	Mm.7996	fibroblast growth factor 12	Fgf12	5.6
1456395_at	Mm.259072	peroxisome proliferative activated receptor, gamma, coactivator 1 alpha	Ppargc1a	5.55
1420653_at	Mm.248380	transforming growth factor, beta 1	Tgfb1	4.15
1422982_at	Mm.439657	androgen receptor	Ar	3.33
1418093_a_at	Mm.252481	epidermal growth factor	Egf	3.27
1419086_at	Mm.46053	fibroblast growth factor binding protein 1	Fgfbp1	3.12
1420915_at	Mm.277406	signal transducer and activator of transcription 1	Stat1	3.08
1439556_at	Mm.4974	neural cell adhesion molecule 1	Ncam1	2.94
1422397_a_at	Mm.200196	interleukin 15 receptor, alpha chain	Il15ra	2.7
1425620_at	Mm.200775	transforming growth factor, beta receptor III	Tgfbr3	2.36

doi:10.1371/journal.pone.0042987.t002

expressed in the inner ear including Oc90 (x131); Tecta (x73); Tectb (x59), Jag1 (x7) and Hes5 (x6) for up P3-list and, GFAP (x120), Kcnj16 (x21) and Gh (x89) for up adult list. In addition, we identified new genes not previously known to be expressed in the mammalian cochlea such as, Hmga2 (x26) and Nrarp (x3) from the up P3 list, and Prl (x460;) and Ar (x3) from the up adult list. For all analyzed genes, the Q-PCR results showed the same variation of gene expression changes as revealed by the microarray approach. However, the fold change values were observed to be mainly greater in Q-PCR when compared to those obtained from the microarrays. The differential expression revealed by Q-PCR was significant for 17 of 18 up P3 analyzed genes (94%) (p-value <0.05). In the case of adult up-regulated genes, the changes in the expression levels were significant for 15 of 20 genes (p-value <0.05) (75%) (Fig. 3).

In addition, to confirm the changes in expression at the protein level (Fig. 4), we performed immunocytochemistry for a newly identified gene (i.e., Hmga2) known to be important for chromatin remaniement and pluripotency in stem cells [33]. We observed up-regulation of Hmga2 within the P3 CSE in a variety of supporting cell subtypes (i.e., Dieters' cells, border cells, inner and outer pillar cells and Hensen's cells). The hair cells showed a relatively weak Hmga2 immunoreaction as compared to that of the surrounding supporting cells.

Functional Annotation of Selected Genes Differentially Expressed between P3 and Adult CSE

IPA was used to access to functional properties of the selected genes up-regulated in P3 and adult CSE (Tables 1 and 2), confirmed by Q-PCR. Only significant functions ($p<0.001$) were reported. The selected P3 up-regulated genes were associated with multiple functions including auditory and vestibular system development, nervous system development, cellular growth/proliferation, and embryonic development (Fig. S1A) and signaling pathways such as, Notch signaling, embryonic stem cell pluripotency and Wnt/βcatenin signaling (Fig. S1B). Among these pathways, four are above threshold for significance ($p<0.05$). Inversely, the adult CSE up-regulated genes were related to biological functions including cell-to-cell interactions, DNA replication, cell cycle and cell death (Fig. S1C). For the pathways, the 26 selected up-regulated adult genes are mainly associated to NF-kB signaling, glucorticoid receptor signaling, FGF signaling and actin cytoskeleton signaling (Fig. S1D). DAVID (Database for Annotation, Visualization and Integrated Discovery) analysis gave mainly similar results as reported by IPA function and pathway analysis (data not shown). Gene lists from comparisons showing significant differences in gene expression were submitted to DAVID) (www.david.abcc.ncifcrf.gov). DAVID provides exploratory visualization tools that promote discovery through functional

IMAGE REMOVED

Figure 1. A schematic summary of the postnatal day-3 and adult cochlear dissection protocol. Cochleae were extracted from P3 and adult mouse inner ears. The otic capsule, the modiolus, the spiral ligament, the stria vascularis and the Reissner's membrane were removed. The tectorial membrane was partially removed from the surface of the epithelia. The cochlear sensory epithelia (CSE) were collected for transcriptomic analysis. Cross-sections depict cell subtypes included in cell preparations harvested from P3 and adult CSE. All samples are mostly comprised of tissue from the three cochlear turns. Scheme is not to scale. IHC: inner hair cells; OHC: outer hair cells; HeC: Hensen cells; BC: border cells; IPC: inner phalengeal cells; PC: pillar cells; DC: Deiter's cells; BM: basilar membrane; IDC: interdental cells; GER: greater epithelial ridge; LER: lesser epithelial ridge; ISC: inner sulcus cells.
doi:10.1371/journal.pone.0042987.g001

classification while simultaneously remaining linked to rich sources of biological annotation.

Potential Networks Associated with P3 Specific Genes

IPA network analysis on the top 20 up P3 genes identified three significant networks. The Figure 5 illustrates the number one ranked network. Most of selected up P3 genes, 17 of 20, interact with each other within this network. These genes are mostly related to cochlea and nervous system development and function. Notch signaling pathway appears notably in this network. It is represented by Hes5, Hey1, Jag1, Atoh1 and, also the new identified Notch signaling transcript in the CSE (i.e., Nrarp). The network shows interactions within Notch pathway and also between Notch players and other genes known in the early development of cochlea (i.e., Sox2, Tgfb and Fgf3). Hmga2, a newly identified gene in P3 mouse CSE, is involved in stem cell pluripotency in other tissues, appears among the genes within this network. However, it does not exhibit direct interactions with known cochlear stem/progenitor cells markers (i.e., Sox2, Jag1).

In order to gain insights on the potential function of Hmga2 in the P3 CSE, we explored the interactions between up-regulated genes included in the 1000 P3 list by using the IPA software. Network with 1000 genes showed more interactions as compared to 20 genes. The network involving Hmga2 is illustrated in Figure 6. Most of the genes forming this network are associated with epigenetic modifications of DNA, such as HDAC2 and Smarce. These interactions are concordant with a role of Hmga2 as a DNA binding protein that alters the architecture of chromatin to enhance gene transcription. The network (Fig. 6) shows two direct interactions for Hmga2: Let7microRNA-Hmga2 and Hmga2-HDAC2 interactions. Interestingly, both MirLet7 and HDAC2 have been shown to have a role in newt and bird inner ear regeneration [34,35].

Potential Networks Associated with Adult Specific Genes

Network analysis assembled three significant networks from selected adult up-regulated genes. We present here the highest score network (Fig. 7). This network shows interactions between the most regulated genes in the adult CSE, the Prl (FC>460) and other up regulated adult genes from the selected list such as Gh, Ar and Stat1.

Of interest, the Gh exhibits a greater than 89 FC increase in adult CSE. As shown in (Figs. S2, S3), both Prl and Gh signaling pathways act via Jak2-Stat pathway involving Stat transcription factors. This may explain the interactions of Prl and Gh with Stat genes in the network shown in Figure 7. Interestingly, Stat1 has been demonstrated to be involved in cisplatin-mediated apoptosis of HCs within the cochlea, attenuated by the use of siRNA against Stat1 [36]. The up-regulation of Stat1 (FC>3) in adult CSE suggests that it may have a role in age-related HC death in the adult cochlea.

Within the network (Fig. 7), both Prl and Gh displayed interactions with the Ar, which previously was not reported to be expressed in the mammalian cochlea. Also, in the same network, both Prl and Gh interact with the Ap1 complex. Ap1 is a dimer formed by c-Jun and c-Fos, that acts as a transcription factor in several functions such as, apoptosis and proliferation. In the cochlea, the up-regulation of Ap1 has been suggested to be involved in HC death after either noise trauma or ototoxic drug exposure [37,38].

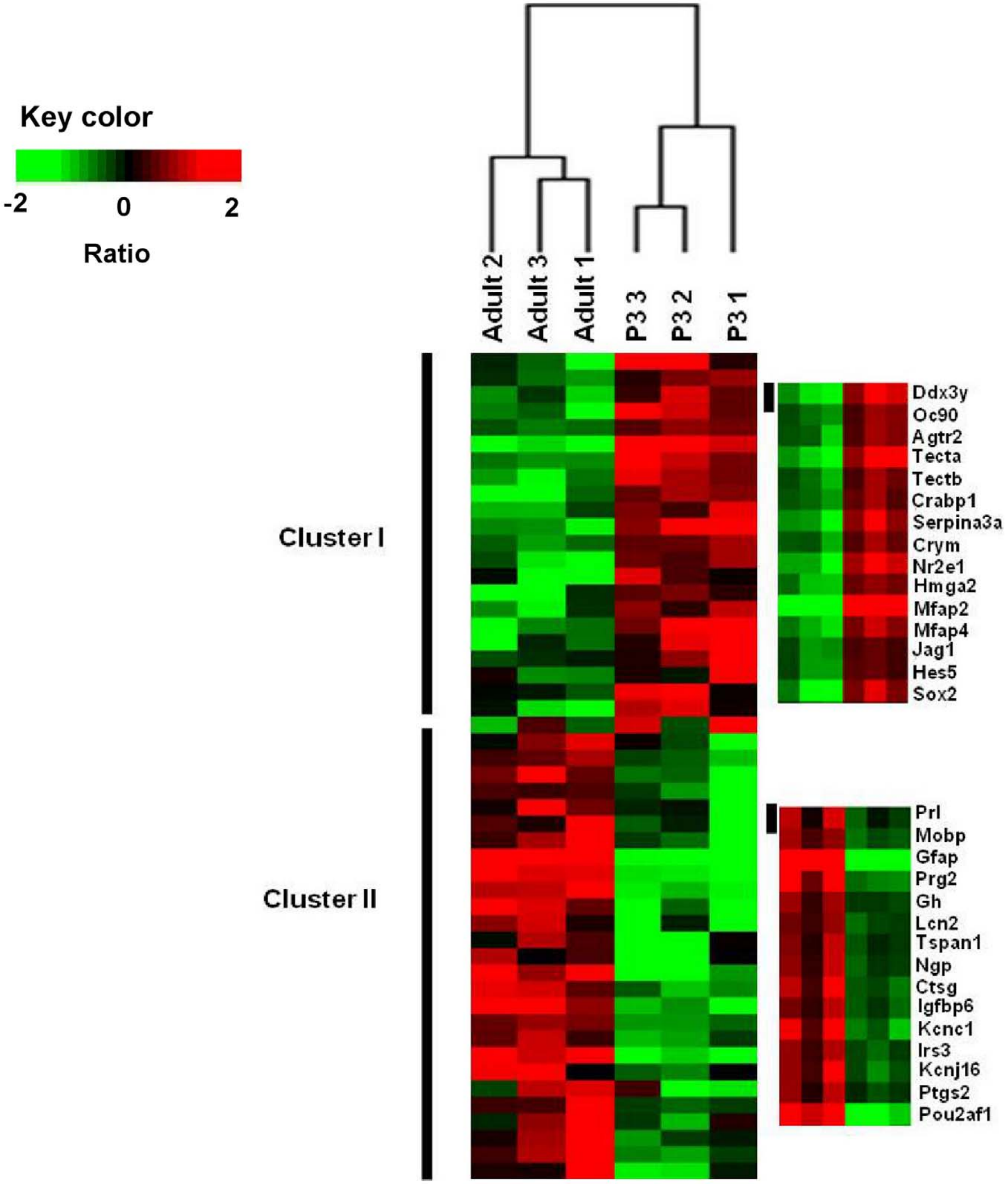

Figure 2. Hierarchical clustering. The expression signature of P3 and adult CSE were visualized by hierarchical clustering on the 5,644 significantly regulated probe sets. The six samples are arranged in columns and transcripts are arranged in rows. The transcripts segregate into two main major clusters. As observed in dendrogram, a first branch indicates a clear segregation between the two stages (All the replicate samples of the P3 group self-cluster into one branch. Adult samples self-cluster into another branch into which all the samples self-cluster). In each stage, a tree represents relationship among samples whose branch lengths reflect the degree of similarity between the samples according to gene expression profile (The samples with similar expression patterns are adjacent). Genes up-regulated in and down-regulated in each group were color-coded in red and green respectively. Cluster I regroups the P3 up-regulated transcripts (i.e., Jag1, Hes5, Sox2). Cluster II was a group of transcripts up-regulated in the adult cochlea samples (i.e., Prl, Gh, Gfap).
doi:10.1371/journal.pone.0042987.g002

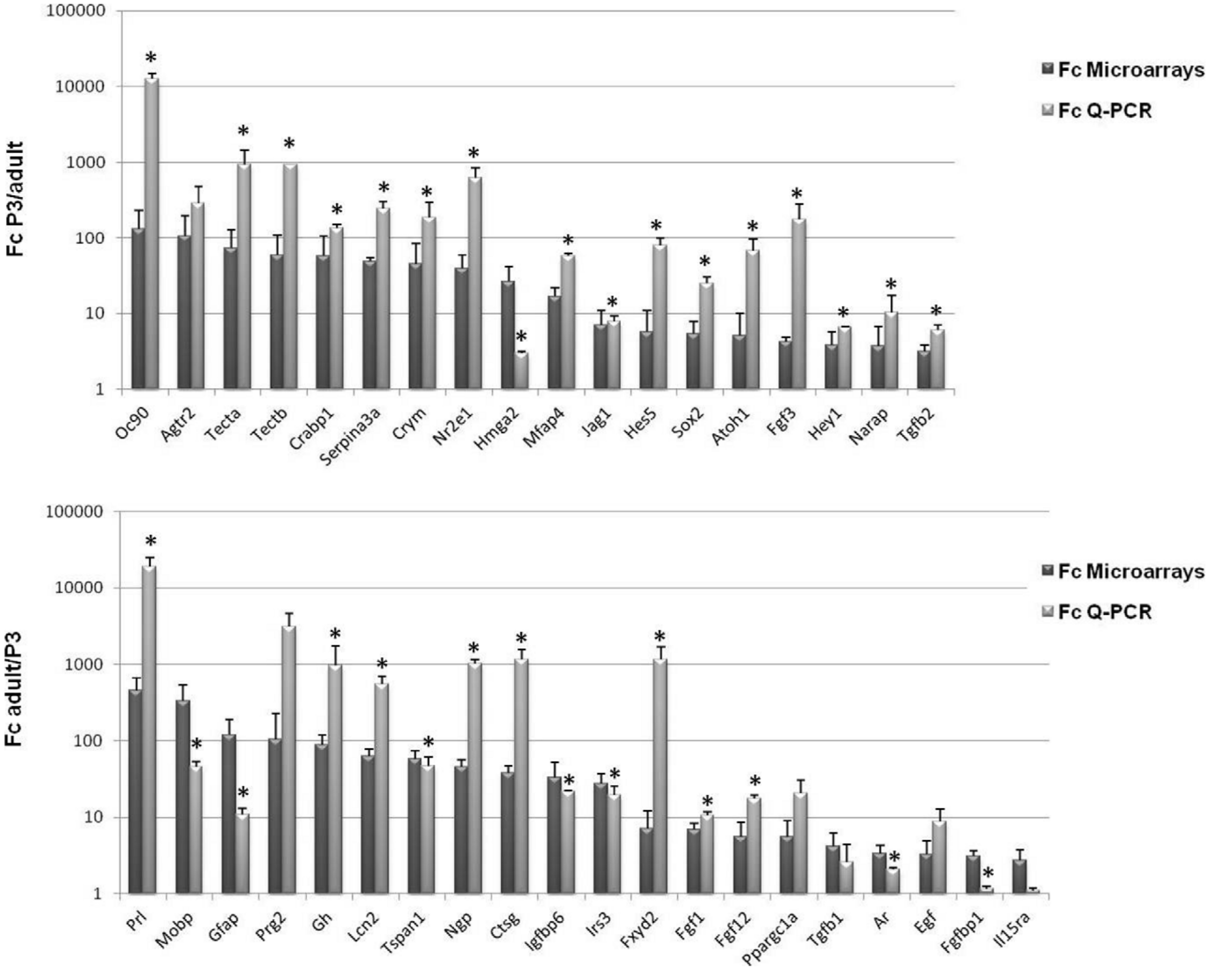

Figure 3. Validation of microarrays results by Q-PCR. A set of selected P3 up-regulated (**A**) and adult up-regulated genes (**B**) were analyzed by real-time Q-PCR to validate the microarray data. All of the Q-RT-PCR results were normalized to the expression of GAPDH in each sample. A Mann Whitney test was performed on Q-PCR results. * indicates significant p value <0.05.
doi:10.1371/journal.pone.0042987.g003

Discussion

In this study, we report for the first time a large-scale analysis of changes in gene expression of CSE dissected from P3 and adult mice. The results of this approach may provide directions for future investigations into the understanding of the known difference in the ability for regeneration/repair between the early postnatal/developing and adult cochleae. We detected a number of genes that were known in the P3 (i.e., Jag1, Hey1, Sox2) and adult (i.e., Gfap, Kcnj16, Gh). In addition, we detected unreported genes in the P3 (i.e., Nrarp, Hmga2) and adult (i.e., Ar and Prl) mouse cochleae. These genes have been shown to be linked into known networks and pathways not previously implicated in the mammalian cochlea. We will then focus principally on these new genes regulated between P3 and adult CSE and their respective networks to illustrate the gene expression regulation.

In the past decade, acumulating evidence has suggested essential roles for Notch and Wnt/β-catenin signaling in the vertebrates inner ear development [39,40]. Our gene analysis revealed more than 3-fold change in the expression of Nrarp (Notch-regulated ankyrin repeat protein), an element of a negative feedback system that attenuates Notch pathway-mediated signaling [41] that has not been previously reported in the inner ear. An increase in the Nrarp transcription has been observed following the induction of Notch signaling, suggesting its involvement in the Notch signaling inhibitory feedback loop [42]. The expression of the Nrarp in the P3 CSE suggests that alternate Notch signaling pathways are operating to pattern the developing CSE in a context dependent manner. Further work is needed to elucidate the specific role of this Notch1 regulatory molecule in the mammalian cochlea that may help to shed more light on the diverse roles of the Notch signaling during inner ear development [43].

The stem cell pluripotency is amongst the most significant pathways related to P3 CSE. This pathway is principally represented by Sox2, Tgfb2 and Hmga2 in our selected up P3 list. The Hmga2 encodes a small chromatin-associated protein that cooperates with other factors to regulate gene expression [44]. During embryogenesis, the expression of Hmga2 is initially at a maximum level throughout the whole embryo, then is subsequently restricted to mesenchymal derivatives only, with later

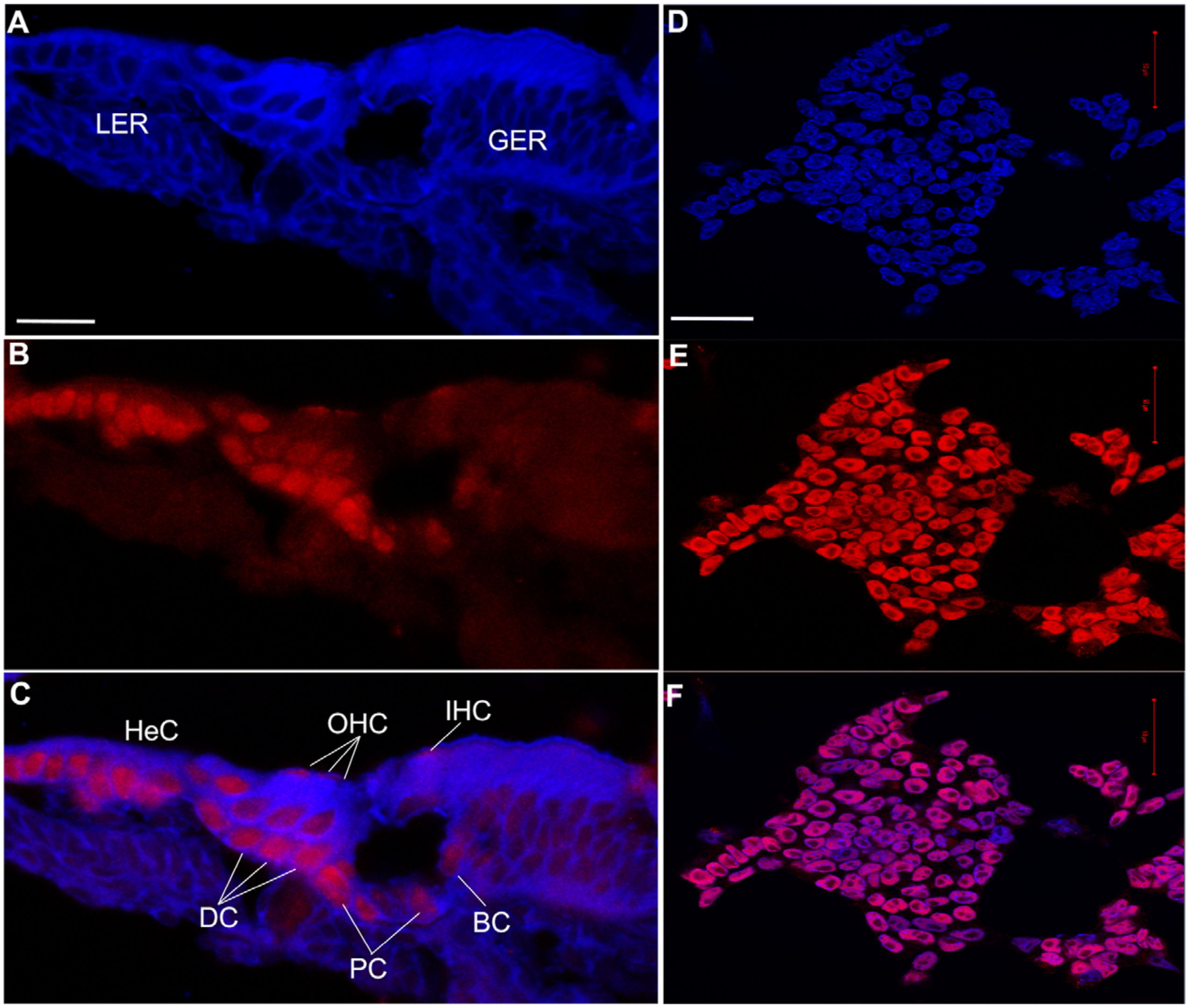

Figure 4. Immunohistochemistry analysis of Hmga2. Photomicrographs of: (A–C) Cross-sections through the middle turn of the P3 cochlea and (D–F) undifferentiated CGR8 mouse embryonic stem (ES) cells growing in feeder free (leukemia inhibitory factor containing medium) cell culture used as positive control for Hmga2 expression. In the P3 cochlea, the expression of the Hmga2 protein (red label) is mostly detected in the supporting cells within the IHC (BC) and OHC (DC) areas, in addition to pillar cells (PC) and Hensen's cells (HeC). The OHCs are weakly labelled with the Hmga2 antiserum. The ES cells and cross-sections are counterstained with DAPI (shown in blue). IHC: inner hair cells; OHC: outer hair cells; BC: border cells; DC: Deiters' cells; GER: greater epithelial ridge; LER: lesser epithelial ridge. Scale bars = 25 µm in (A–C) and 50 µm in (D–F).
doi:10.1371/journal.pone.0042987.g004

expression becoming undetectable in adult tissues [45]. It has been reported that Hmga2 is under the time-dependent regulation of the microRNA let-7 [46]. The let-7 miRNA family was among the first group of miRNA suggested to regulate 'stemness' by repressing self-renewal in both normal development and cancer [47]. Interestingly, Let-7 miRNA members have been suggested as potential regulators of the dedifferentiation in lens and inner ear HC regeneration of the newt [34]. In the same manner, in teleost fish a robust regenerative response to retinal injury relies on Müller glia dedifferentiation into a cycling population of progenitor cells through let-7 miRNA down-regulation [48]. Hmga2 expression is present in human and mouse pluripotent embryonic stem (ES) cells indicating a critical role of these proteins during development and growth [49]. Furthermore, it has been found that Hmga2 expression in human ES cells is closely correlated to the expression of pluripotency specific genes such as, Utf1, Sox2 and Oct4. In addition, it has been shown that Hmga2 promotes neural stem cell self-renewal in young but not old mice by reducing the expression of two negative regulators of the cell cycle, i.e. p16(Ink4a)/p19(Arf) [50]. Interestingly, our microarray showing an up-regulation of Hmga2 in the P3 CSE supports the previously observation as to the persistence of multipotent stem cells in the developing P3 cochlea and their lack in the adult cochlea of the mice [12]. In addition, we have previously demonstrated the expression of a battery of stem/progenitor markers within the P3 CSE and their down-regulation from the adult CSE [14]. The up-regulation of Hmga2 in the P3 cochlea is another stem cell marker that may reaffirm the persistence of multipotent stem cells in the P3 CSE cochlea and their lack in the cochleae of adult mice. The IPA network for P3 CSE upregulated genes (Fig. 6) indicates that Hmga2 directly interacts with HDAC2 (histone diacetylase 2). Interestingly, the HDAC has been demonstrated to regulate supporting cell proliferation during the regenerative proliferation in the avian utricular sensory epithelium

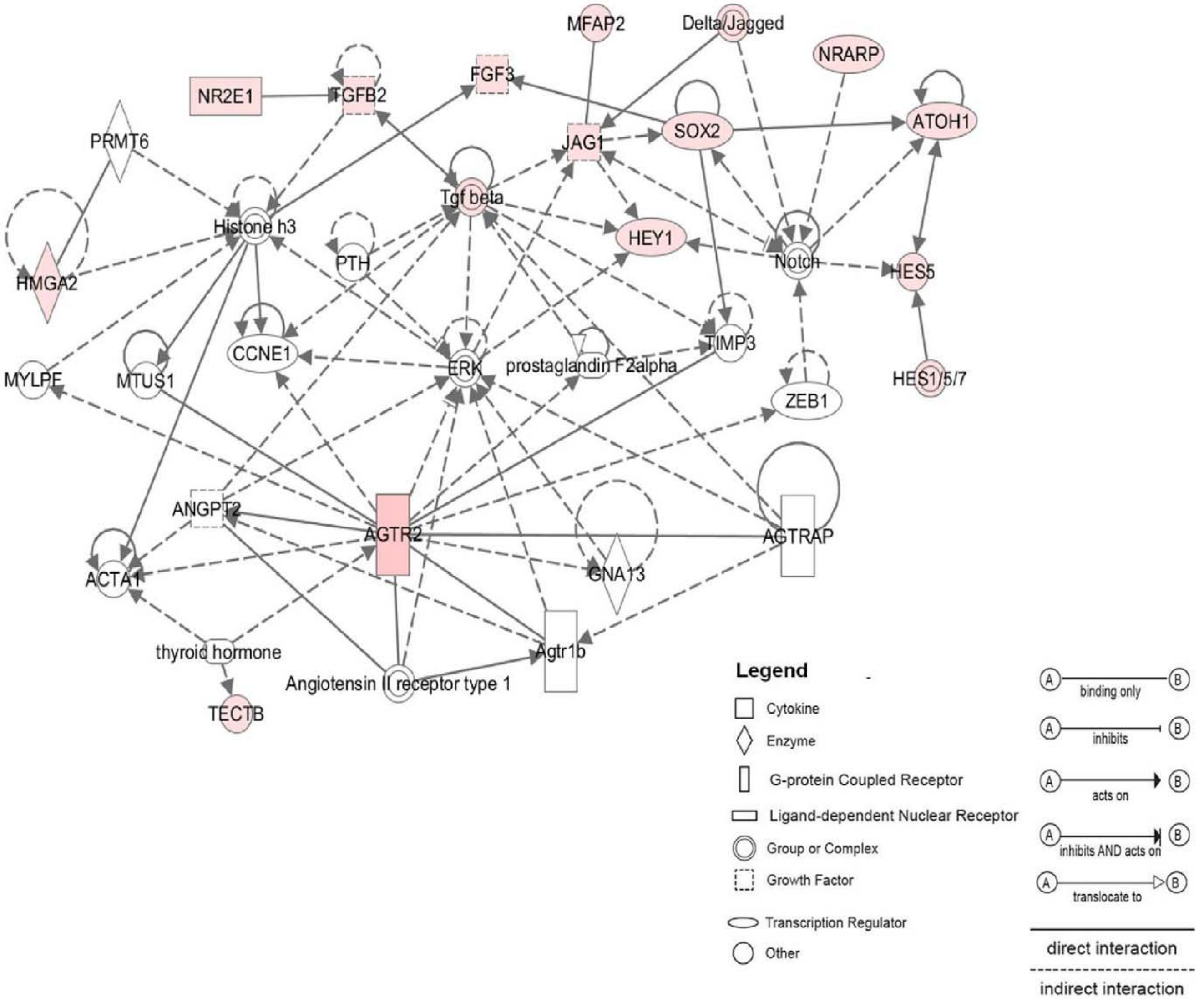

Figure 5. Functional network analysis related to P3 up-regulated genes. The most significant network assembled by selected P3 up-regulated genes includes 15 genes related to inner ear development most of them are components of the Notch pathway (i.e., Hey1, Hes5, Jag1). Hmga2, a newly identified stem cell gene in the P3 CSE appears also among the interacting genes within the network. Solid lines between nodes indicate direct molecular interaction between connected genes whereas dotted lines indicate an indirect functional interaction. Genes included in the P3 signature are in pink and those not found in the signature are in white.
doi:10.1371/journal.pone.0042987.g005

[35]. In another study with the mouse cochlea, HDAC was hypothesized to have a role in aminoglycoside antibiotic-induced HC death since its expression showed a transient increase after gentamicin administration [51]. In another hand, analysis of the selected adult up-regulated genes indicated that the biological functions are mostly related to cell death, polypeptide hormones, cellular growth, and inflammatory/immune response. Of interest, among the 26 selected up adult genes, microarray analysis and Q-PCR validation revealed two secreting pituitary hormones, i.e, growth hormone and prolactin (Gh/Prl). The Prl was the highest expressed gene in the adult CSE with a FC >460. Previous findings have suggested a potential role of Prl in inner ear function. For example, a hyperprolactenemia has been reported in patients presenting with an inner ear dysfunction [52,53]. These observations were followed by a study demonstrating that hyperprolactenemia induced by a long-term estrogen treatment in guinea pig led to conductive hearing loss probably linked to an inner ear ionic homeostasis defect [54]. A recent study showed that hormone replacement therapy involving estrogen and progestin can promote hearing loss supporting the previous data [55]. Our gene expression data are consistent with these findings revealed for the first time that the Prl transcripts are expressed in the adult CSE supporting a role for Prl hormone in auditory function.

Gh was also observed to be among the most significant up-regulated genes in the adult CSE (FC >89). Interestingly, a previous microarray analysis of noise-exposed zebrafish showed that Gh was significantly up-regulated during the process of zebrafish inner ear HC regeneration [20]. In a parallel study, these authors showed that exogenous Gh promotes post-acoustic trauma HC regeneration in the zebrafish ear through the stimulation of cell proliferation [56]. These findings are in contrast with our present data with the mammalian cochlea showing that Gh is endogenously up-regulated in the adult that is known to lack any regenerative capacity [57]. In addition, previous gene expression

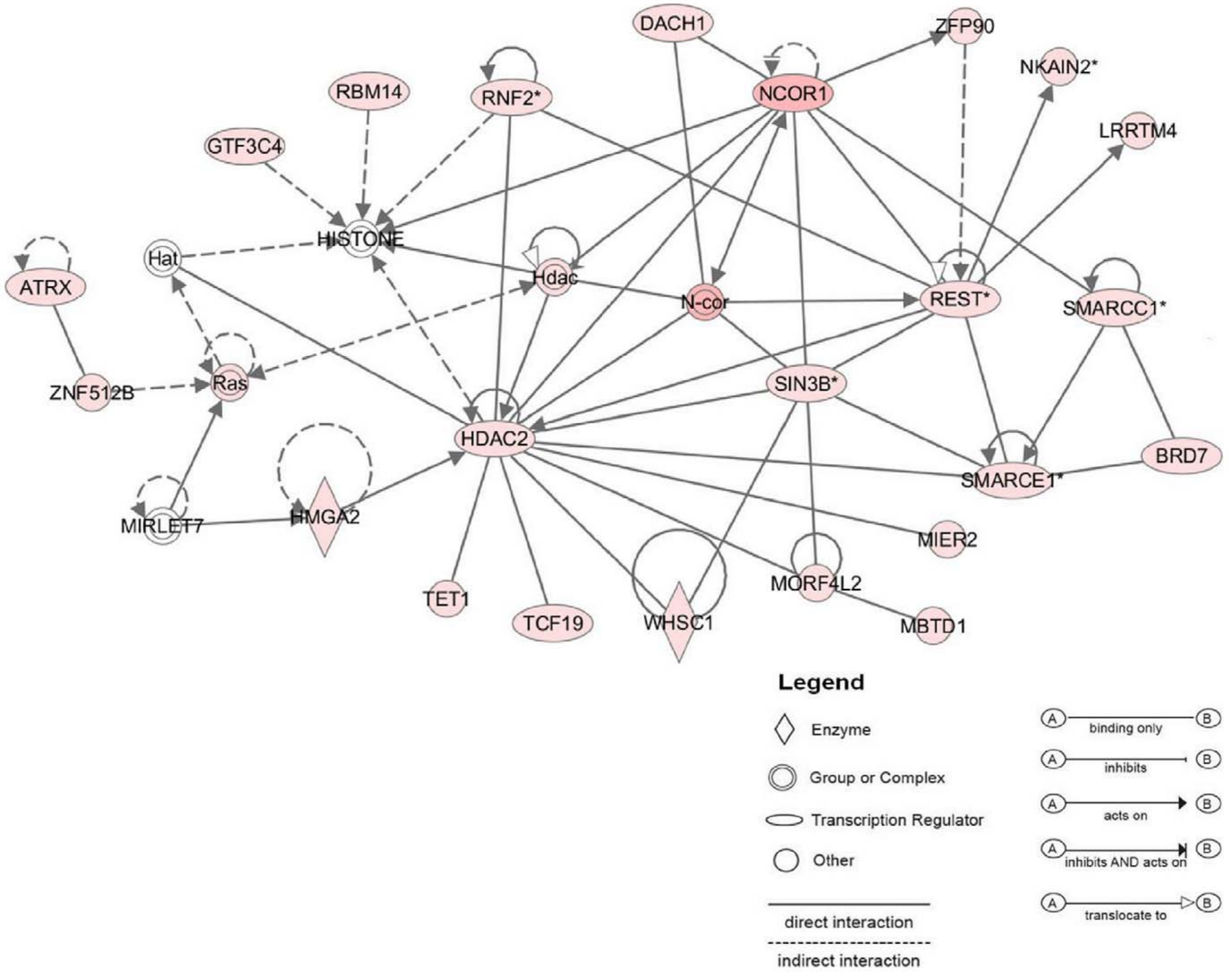

Figure 6. The most significant IPA network assembled around Hmga2 in P3 CSE. IPA results showing the network of Hmga2 and their close interactions with HDAC2 and MIR-Let7 genes. Genes included in the P3 signature are in pink and thouse not found in the signature are in white.
doi:10.1371/journal.pone.0042987.g006

profiling following a noise trauma indicated a two-fold decrease in Gh in the adult rat cochlea [58]. They are also in contrast with the up-regulation of Gh after noise exposition in the zebrafish model. It is possible that the relatively simple structure of the ears of non-mammalian vertebrates combined with spontaneous cell cycle re-entry, is likely to enhance the proliferative promoting action of Gh than can occur in the highly specialized mammalian organ of Corti that relies on a small number of post-mitotic, highly specialized cells for hearing sensitivity. However, the converging point between the studies using the zebrafish and rodent models is the important role of GH in the sensori-neural structures of the inner ear that appears to be conserved across vertebrates and therefore warrants further investigation. We also found that the Androgen receptor (Ar) is among the up-regulated genes in the adult CSE (FC >3). The Ar transcripts have been shown previously to be expressed only in the fish inner ear [59]. Our microarray data demonstrate for the first time the expression of Ar gene in the mammalian cochlea. It has been reported that the Prl and Gh enhance the expression of Ar-mRNA in the rat's prostate cells [60]. This result may explain the synchronic expression of these three transcripts (i.e., Ar, Gh and Prl) in the adult CSE as revealed by our microarray data suggesting that Prl and Gh may be involved in the regulation of Ar-mRNA expression in the adult cochlea. Interestingly, it has been reported in the literature that there is a cross talk between Notch and Ar pathways. Indeed, Hey1, an intracellular effector of the Notch signaling has been shown to act as a specific repressor on Ar expression in mammalian cell lines [61]. Furthermore, gene profiling data showed that the expression of Notch1 and its ligand Jagged1 is regulated by Ar in the prostate [62]. Taken together, these data could explain the down-regulation of Hey1, Notch1, and Jagged1 in the adult CSE where Ar is over-expressed as revealed by our microarray and Q-PCR results.

Overall, we identified a total of 5644 transcripts that significantly (FDR $\leq$ 0.05) distinguished gene expression profile of the P3 and adult CSE. We focused on two highly up-regulated genes (i.e., Nrarp, Hmga2) in postnatal-P3 and two up-regulated genes (i.e., Ar, Prl) in adult CSE as examples to highlight not previously reported genes that may account for the known difference in repair/regenerative ability of the early postnatal and adult mammalian cochleae.

Our data may constitute the first step toward further studies to understand the functional role of regulated and new detected

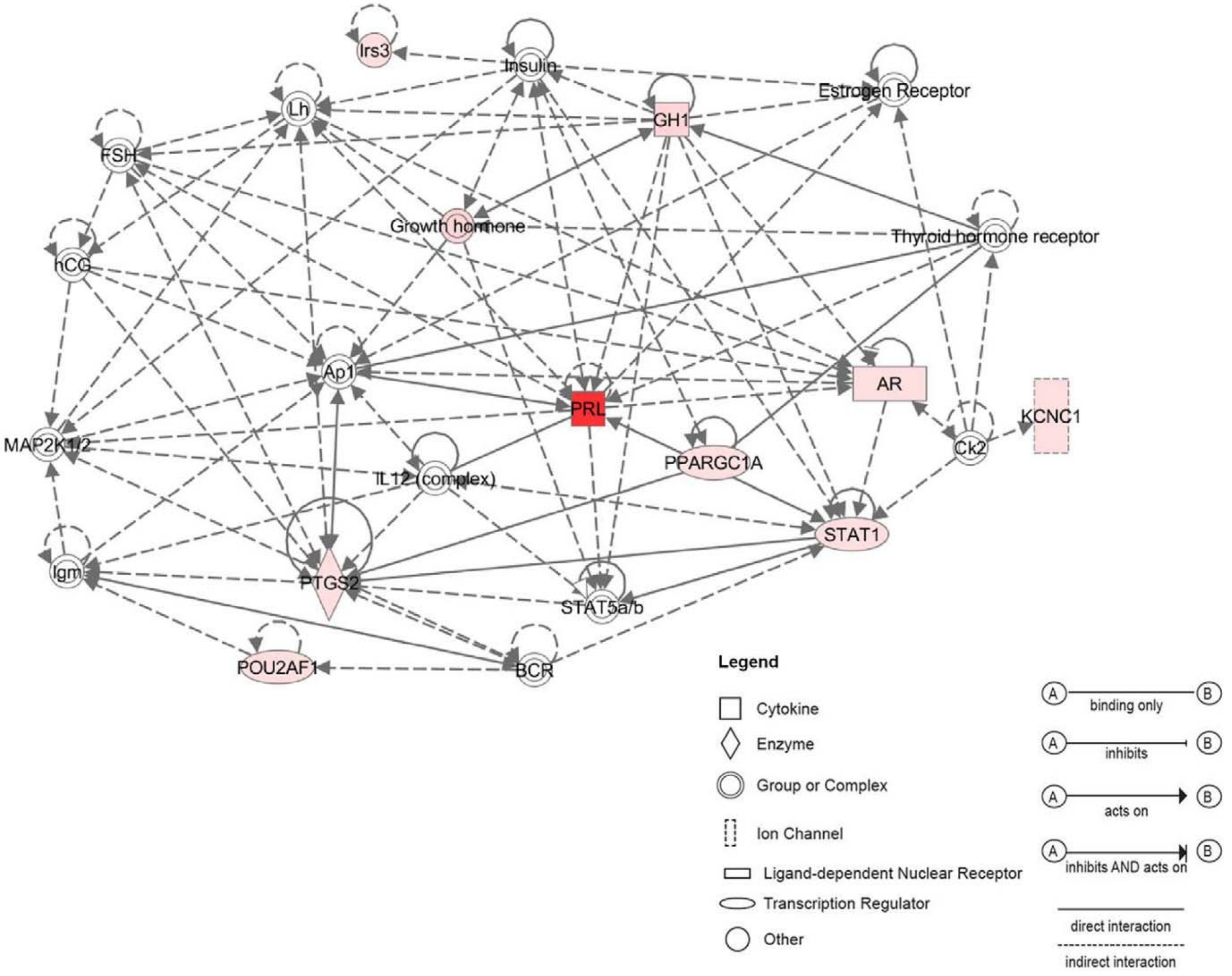

Figure 7. Functional network analysis related to adult up-regulated genes. The most significant network assembled by selected adult CSE up-regulated genes includes 12 genes related to polypeptide hormones (Prl, Gh) and Androgen receptor (Ar). The Prl seems to be a central gene in this network interacting with many molecules including AP1, Stat1 and Stat5.
doi:10.1371/journal.pone.0042987.g007

genes as related to the development and regeneration pathways in the mammalian cochlea.

Supporting Information

Figure S1 Biological functions and pathways associated with the selected differentially expressed genes. (**A**) IPA function analysis on the up-regulated P3 genes. (**B**) IPA pathway analysis on the up-regulated P3 genes. (**C**) IPA function analysis on the up-regulated adult genes. (**D**) IPA Pathways analysis on the up-regulated adult genes. The Threshold represents the p-value = 0.05. The ratio (shown as squares on B and D) represents the number of analyzed genes in a given pathway divided by total number of genes that form this pathway.
(TIF)

Figure S2 Prolactin signaling pathway identified by IPA software. The Prl transduction signal via PRLR shows that Prl may act via Jak2-Stat and/or Ras pathways depending on the function. Jak2-Stat pathway involves Stat1, Stat3 and Stat 5 transcription factors. Ras pathway is activated in cell cycle, proliferation and cell death functions. It involves Ap1 complex in the nucleus formed by c-Jun and c-Fos immediate genes. The pathway figure explains the interactions between Prl, Stat and AP1 shown in the IPA up adult network (Fig. 5).
(TIF)

Figure S3 Growth hormone signaling pathway identified by IPA software. Canonical pathway reveals growth hormone-related gene signaling. Canonical pathway analysis with IPA software showing known growth-hormone related pathways including SOCS, STAT1 transcription factor, and PKC genes. The canonical pathway is established by the IPA software from its previous database of publications and may include computationally generated networks from many tissues at any age. Pink-colored genes are up-regulated genes in the adult list.
(TIF)

Table S1 Primers pairs used for Real time PCR.
(TIFF)

Acknowledgments

We thank Drs T. Van de Water (University of Miami) for his critical reading, B. Biuetiry (University Aix Marseille) for the ES CGR8 cells, M. Narita (Cambridge Institute) for the HMGA2 antibody and A. Dos Santos (UMR 7260, University Aix Marseille) for cell culture. We also thank Dr. P. Ravel (Faculty of Pharmacy, University of Montpellier I) for help with statistical analysis. The authors thank two anonymous reviewers for their critical reading and helpful comments on an earlier version of the manuscript.

Author Contributions

Conceived and designed the experiments: IS AZ. Performed the experiments: IS SA ES. Analyzed the data: IS SA SM AZ. Wrote the paper: IS SA AZ.

References

1. Hudspeth AJ (1997) How hearing happens. Neuron 19: 947–950.
2. Warchol ME (2011) Sensory regeneration in the vertebrate inner ear: Differences at the levels of cells and species. Hear. Res. 273: 72–79.
3. Zine A, de Ribaupierre F (1998) Replacement of mammalian auditory hair cells. Neuroreport 9: 263–268.
4. Staecker H, Van De Water TR (1998) Factors controlling hair-cell regeneration/repair in the inner ear. Curr. Opin. Neurobiol. 8: 480–487.
5. Lenoir M, Vago P (1997) Does the organ of Corti attempt to differentiate new hair cells after antibiotic intoxication in rat pups? Int. J. Dev. Neurosci. 15: 487–495.
6. Chen P, Zindy F, Abdala C, Liu F, Li X, et al. (2003) Progressive hearing loss in mice lacking the cyclin-dependent kinase inhibitor Ink4d. Nat. Cell Biol. 5: 422–426.
7. Sage C, Huang M, Karimi K, Gutierrez G, Vollrath MA, et al. (2005) Proliferation of functional hair cells in vivo in the absence of the retinoblastoma protein. Science 307: 1114–1118.
8. Mantela J, Jiang Z, Ylikoski J, Fritzsch B, Zacksenhaus E, et al. (2005) The retinoblastoma gene pathway regulates the postmitotic state of hair cells of the mouse inner ear. Development 132: 2377–2388.
9. Laine H, Doetzlhofer A, Mantela J, Ylikoski J, Laiho M, et al. (2007) p19 (Ink4d) and p21(Cip1) collaborate to maintain the postmitotic state of auditory hair cells, their codeletion leading to DNA damage and p53-mediated apoptosis. J. Neurosci. 27: 1434–1444.
10. Laine H, Sulg M, Kirjavainen A, Pirvola U (2010) Cell cycle regulation in the inner ear sensory epithelia: role of cyclin D1 and cyclin-dependent kinase inhibitors. Dev. Biol. 337: 134–146.
11. Savary E, Hugnot JP, Chassigneux Y, Travo C, Duperray C, et al. (2007) Distinct population of hair cell progenitors can be isolated from the postnatal mouse cochlea using side population analysis. Stem Cells 25: 332–339.
12. Oshima K, Grimm CM, Corrales CE, Senn P, Martinez Monedero R, et al. (2007) Differential distribution of stem cells in the auditory and vestibular organs of the inner ear. J. Assoc. Res. Otolaryngol. 8: 18–31.
13. Diensthuber M, Oshima K, Heller S (2009) Stem/progenitor cells derived from the cochlear sensory epithelium give rise to spheres with distinct morphologies and features. J. Assoc. Res. Otolaryngol 10: 173–190.
14. Smeti I, Savary E, Capelle V, Hugnot JP, Uziel A, et al. (2011) Expression of candidate markers for stem/progenitor cells in the inner ears of developing and adult GFAP and nestin promoter-GFP transgenic mice. Gene Expr. Patterns 11: 22–32.
15. Trevino V, Falciani F, Barrera-Saldaña HA (2007) DNA microarrays: a powerful genomic tool for biomedical and clinical research. Mol. Med 13: 527–541.
16. Hawkins RD, Bashiardes S, Helms CA, Hu L, Saccone NL, et al. (2003) Gene expression differences in quiescent versus regenerating hair cells of avian sensory epithelia: implications for human hearing and balance disorders. Hum. Mol. Genet 12: 1261–1272.
17. Hawkins RD, Bashiardes S, Powder KE, Sajan SA, Bhonagiri V, et al. (2007) Large scale gene expression profiles of regenerating inner ear sensory epithelia. PLoS ONE 2: e525.
18. Paxton CN, Bleyl SB, Chapman SC, Schoenwolf GC (2010) Identification of differentially expressed genes in early inner ear development. Gene Expr. Patterns 10: 31–43.
19. McDermott BM Jr, Baucom JM, Hudspeth AJ (2007) Analysis and functional evaluation of the hair-cell transcriptome. Proc. Natl. Acad. Sci. U.S.A. 104: 11820–11825.
20. Schuck JB, Sun H, Penberthy WT, Cooper NGF, Li X, et al. (2011) Transcriptomic analysis of the zebrafish inner ear points to growth hormone mediated regeneration following acoustic trauma. BMC Neurosci 12: 88.
21. Lin J, Ozeki M, Javel E, Zhao Z, Pan W, et al. (2003) Identification of gene expression profiles in rat ears with cDNA microarrays. Hear. Res 175: 2–13.
22. Cho Y, Gong T-WL, Stöver T, Lomax MI, Altschuler RA (2002) Gene expression profiles of the rat cochlea, cochlear nucleus, and inferior colliculus. J. Assoc. Res. Otolaryngol 3: 54–67.
23. Morris KA, Snir E, Pompeia C, Koroleva IV, Kachar B, et al. (2005) Differential expression of genes within the cochlea as defined by a custom mouse inner ear microarray. J. Assoc. Res. Otolaryngol 6: 75–89.
24. Sajan SA, Warchol ME, Lovett M (2007) Toward a systems biology of mouse inner ear organogenesis: gene expression pathways, patterns and network analysis. Genetics 177: 631–653.
25. Sato T, Doi K, Hibino H, Kubo T (2009) Analysis of gene expression profiles along the tonotopic map of mouse cochlea by cDNA microarrays. Acta Otolaryngol Suppl: 12–17.
26. Chen Z-Y, Corey DP (2002) An inner ear gene expression database. J. Assoc. Res. Otolaryngol 3: 140–148.
27. National Center for Biotechnology Information website. Guide for the Care and Use of Laboratory Animals -NCBI Bookshelf. Available: http://www.ncbi.nlm.nih.gov/books/NBK54050/.
28. Meghan D, Konrad NT (2006) Mapping quantitative trait loci for hearing loss in Black Swiss mice. Hear. Res 212: 128–139.
29. Tusher VG, Tibshirani R, Chu G (2001) Significance analysis of microarrays applied to the ionizing radiation response. Proc. Natl. Acad. Sci. U.S.A. 98: 5116–5121.
30. Eisen MB, Spellman PT, Brown PO, Botstein D (1998) Cluster analysis and display of genome-wide expression patterns. Proc. Natl. Acad. Sci. U.S.A. 95: 14863–14868.
31. Sokal RR, Michener CD (1958) A Statistical Method for Evaluating Systematic Relationships. Univ Kans Sci Bull 38:1409–1438.
32. Livak KJ, Schmittgen TD (2001) Analysis of relative gene expression data using real-time quantitative PCR and the 2(-Delta Delta C(T)) Method. Methods 25: 402–408.
33. Pfannkuche K, Summer H, Li O, Hescheler J, Dröge P (2009) The high mobility group protein HMGA2: a co-regulator of chromatin structure and pluripotency in stem cells? Stem Cell Rev 5: 224–230.
34. Tsonis PA, Call MK, Grogg MW, Sartor MA, Taylor RR, et al. (2007) microRNAs and regeneration: *let-7* members as potential regulators of dedifferentiation in lens and inner ear hair cell regeneration of the adult newt. Biochem Biophys Res Commun. 362:940–945.
35. Slattery EL, Speck JD, Warchol ME (2009) Epigenetic influences on sensory regeneration: histone deacetylases regulate supporting cell proliferation in the avian utricle. J. Assoc. Res. Otolaryngol. 10: 341–353.
36. Kaur T, Mukherjea D, Sheehan K, Jajoo S, Rybak LP, et al. (2011) Short interfering RNA against STAT1 attenuates cisplatin-induced ototoxicity in the rat by suppressing inflammation. Cell Death Dis 2: e180.
37. Ogita K, Matsunobu T, Schacht J (2000) Acoustic trauma enhances DNA binding of transcription factor AP-1 in the guinea pig inner ear. Neuroreport 11: 859–862.
38. Nagashima R, Sugiyama C, Yoneyama M, Ogita K (2005) Transcriptional factors in the cochlea within the inner ear. J. Pharmacol. Sci. 99: 301–306.
39. Fritzsch B, Jahan I, Pan N, Kersigo J, Duncan J, et al. (2011) Dissecting the molecular basis of organ of Corti development: Where are we now? Hear. Res. 276: 16–26.
40. Groves AK, Fekete DM (2012) Shaping sound in space: the regulation of inner ear patterning. Development 139: 245–257.
41. Krebs LT, Deftos ML, Bevan MJ, Gridley T (2001) The Nrarp gene encodes an ankyrin-repeat protein that is transcriptionally regulated by the notch signaling pathway. Dev. Biol. 238: 110–119.
42. Pirot P, van Grunsven LA, Marine J-C, Huylebroeck D, Bellefroid EJ (2004) Direct regulation of the Nrarp gene promoter by the Notch signaling pathway. Biochem. Biophys. Res. Commun. 322: 526–534.
43. Daudet N, Lewis J (2005) Two contrasting roles for Notch activity in chick inner ear development: specification of prosensory patches and lateral inhibition of hair-cell differentiation. Development 132: 541–551.
44. Merika M, Thanos D (2001) Enhanceosomes. Curr. Opin. Genet. Dev. 11: 205–208.
45. Hirning-Folz U, Wilda M, Rippe V, Bullerdiek J, Hameister H (1998) The expression pattern of the Hmgic gene during development. Genes Chromosomes Cancer 23: 350–357.
46. Mayr C, Hemann MT, Bartel DP (2007) Disrupting the pairing between let-7 and Hmga2 enhances oncogenic transformation. Science 315: 1576–1579.
47. Büssing I, Slack FJ, Grosshans H (2008) let-7 microRNAs in development, stem cells and cancer. Trends Mol Med 14: 400–409.
48. Ramachandran R, Fausett BV, Goldman D (2010) Ascl1a regulates Müller glia dedifferentiation and retinal regeneration through a Lin-28-dependent, let-7 microRNA signalling pathway. Nat. Cell Biol. 12: 1101–1107.
49. Li O, Li J, Dröge P (2007) DNA architectural factor and proto-oncogene HMGA2 regulates key developmental genes in pluripotent human embryonic stem cells. FEBS Lett. 581: 3533–3537.
50. Nishino J, Kim I, Chada K, Morrison SJ (2008) Hmga2 promotes neural stem cell self-renewal in young but not old mice by reducing p16Ink4a and p19Arf Expression. Cell 135: 227–239.
51. Chen F-Q, Schacht J, Sha S-H (2009) Aminoglycoside-induced histone deacetylation and hair cell death in the mouse cochlea. J. Neurochem. 108: 1226–1236.

52. Horner KC, Guieu R, Magnan J, Chays A, Cazals Y (2002) Prolactinoma in some Ménière's patients–is stress involved? Neuropsychopharmacology 26: 135–138.
53. Falkenius-Schmidt K, Rydmarker S, Horner KC (2005) Hyperprolactinemia in some Meniere patients even in the absence of incapacitating vertigo. Hear. Res 203: 154–158.
54. Horner KC, Cazals Y, Guieu R, Lenoir M, Sauze N (2007) Experimental estrogen-induced hyperprolactinemia results in bone-related hearing loss in the guinea pig. Am. J. Physiol. Endocrinol. Metab 293: E1224–1232.
55. Price K, Zhu X, Guimaraes PF, Vasilyeva ON, Frisina RD (2009) Hormone replacement therapy diminishes hearing in peri-menopausal mice. Hear. Res 252: 29–36.
56. Sun H, Lin C-H, Smith ME (2011) Growth hormone promotes hair cell regeneration in the zebrafish (Danio rerio) inner ear following acoustic trauma. PLoS ONE 6: e28372.
57. Kopecky B, Fritzsch B (2011) Regeneration of Hair Cells: Making Sense of All the Noise. Pharmaceuticals. 4: 848–879.
58. Cho Y, Gong T-WL, Kanicki A, Altschuler RA, Lomax MI (2004) Noise overstimulation induces immediate early genes in the rat cochlea. Brain Res. Mol. Brain Res. 130: 134–148.
59. Forlano PM, Marchaterre M, Deitcher DL, Bass AH (2010) Distribution of androgen receptor mRNA expression in vocal, auditory, and neuroendocrine circuits in a teleost fish. J. Comp. Neurol 518: 493–512.
60. Reiter E, Bonnet P, Sente B, Dombrowicz D, de Leval J, et al. (1992) Growth hormone and prolactin stimulate androgen receptor, insulin-like growth factor-I (IGF-I) and IGF-I receptor levels in the prostate of immature rats. Mol. Cell. Endocrinol 88: 77–87.
61. Belandia B, Powell SM, García-Pedrero JM, Walker MM, Bevan CL, et al. (2005) Hey1, a mediator of notch signaling, is an androgen receptor corepressor. Mol. Cell. Biol 25: 1425–1436.
62. Nantermet PV, Xu J, Yu Y, Hodor P, Holder D, et al. (2004) Identification of genetic pathways activated by the androgen receptor during the induction of proliferation in the ventral prostate gland. J. Biol. Chem 279: 1310–1322.
